# Kinetics of Bovine leukemia virus aspartic protease reveals its dimerization and conformational change

**DOI:** 10.1371/journal.pone.0271671

**Published:** 2022-07-22

**Authors:** Martín Fló, Federico Carrión, Natalia Olivero-Deibe, Sergio Bianchi, Madelón Portela, Florencia Rammauro, Beatriz Alvarez, Otto Pritsch

**Affiliations:** 1 Laboratorio de Inmunovirología, Institut Pasteur de Montevideo, Montevideo, Uruguay; 2 Departamento de Inmunobiología, Facultad de Medicina, Universidad de la República, Montevideo, Uruguay; 3 Laboratorio de Biomarcadores Moleculares, Departamento de Fisiopatología, Hospital de Clínicas, Facultad de Medicina, Universidad de la República, Montevideo, Uruguay; 4 Unidad de Bioquímica y Proteómica Analíticas, Institut Pasteur de Montevideo, Facultad de Ciencias, Montevideo, Uruguay; 5 Laboratorio de Enzimología, Instituto de Química Biológica, Facultad de Ciencias, Universidad de la República, Montevideo, Uruguay; Consejo Superior de Investigaciones Cientificas, SPAIN

## Abstract

The retropepsin (PR) of the Bovine leukemia virus (BLV) plays, as in other retroviruses, a crucial role in the transition from the non-infective viral particle to the infective virion by processing the polyprotein Gag. PR is expressed as an immature precursor associated with Gag, after an occasional −1 ribosomal frameshifting event. Self-hydrolysis of PR at specific N- and C-terminal sites releases the monomer that dimerizes giving rise to the active protease. We designed a strategy to express BLV PR in *E*. *coli* as a fusion protein with maltose binding protein, with a six-histidine tag at its N-terminal end, and bearing a tobacco etch virus protease hydrolysis site. This allowed us to obtain soluble and mature recombinant PR in relatively good yields, with exactly the same amino acid composition as the native protein. As PR presents relative promiscuity for the hydrolysis sites we designed four fluorogenic peptide substrates based on Förster resonance energy transfer (FRET) in order to characterize the activity of the recombinant enzyme. These substrates opened the way to perform kinetic studies, allowing us to characterize the dimer-monomer equilibrium. Furthermore, we obtained kinetic evidence for the existence of a conformational change that enables the interaction with the substrate. These results constitute a starting point for the elucidation of the kinetic properties of BLV-PR, and may be relevant not only to improve the chemical warfare against this virus but also to better understand other viral PRs.

## Introduction

*Bovine leukemia virus* (BLV), together with *Human T-cell leukemia virus* (HTLV-I, HTLV-II y HTLV-III) and *Simian T-cell leukemia virus* (STLV-I, STLV-II y STLV-III), comprises the genus *Deltaretrovirus* within the *Retroviridae* family according to International Committee on Taxonomy of Viruses ICTV (https://talk.ictvonline.org/). BLV is the etiological agent of a disease that mainly affects dairy cattle called enzootic bovine leukosis [1]. This disease is characterized by a prolonged incubation, and a chronic and often subclinical course. Most (60 to 70%) of the animals infected with BLV are asymptomatic carriers of the virus, 20 to 30% develop a benign polyclonal proliferation of CD5^+^ B cells called persistent lymphocytosis, and 5–10% develop an aggressive tumor pathology (lymphosarcoma) [2–4]. BLV is distributed worldwide in all continents, except in some areas where BLV-free status has been achieved after great efforts to implement control measures and eradication campaigns [5–7]. The epidemiological presentation of the disease produces economic losses in the agricultural sector. In fact, there is a drop in productivity and profitability even in the context of subclinical BLV infection. Annual global losses in milk production caused by BLV infection are estimated to represent 2.5 to 3.5% [8, 9].

**Fig 1:**
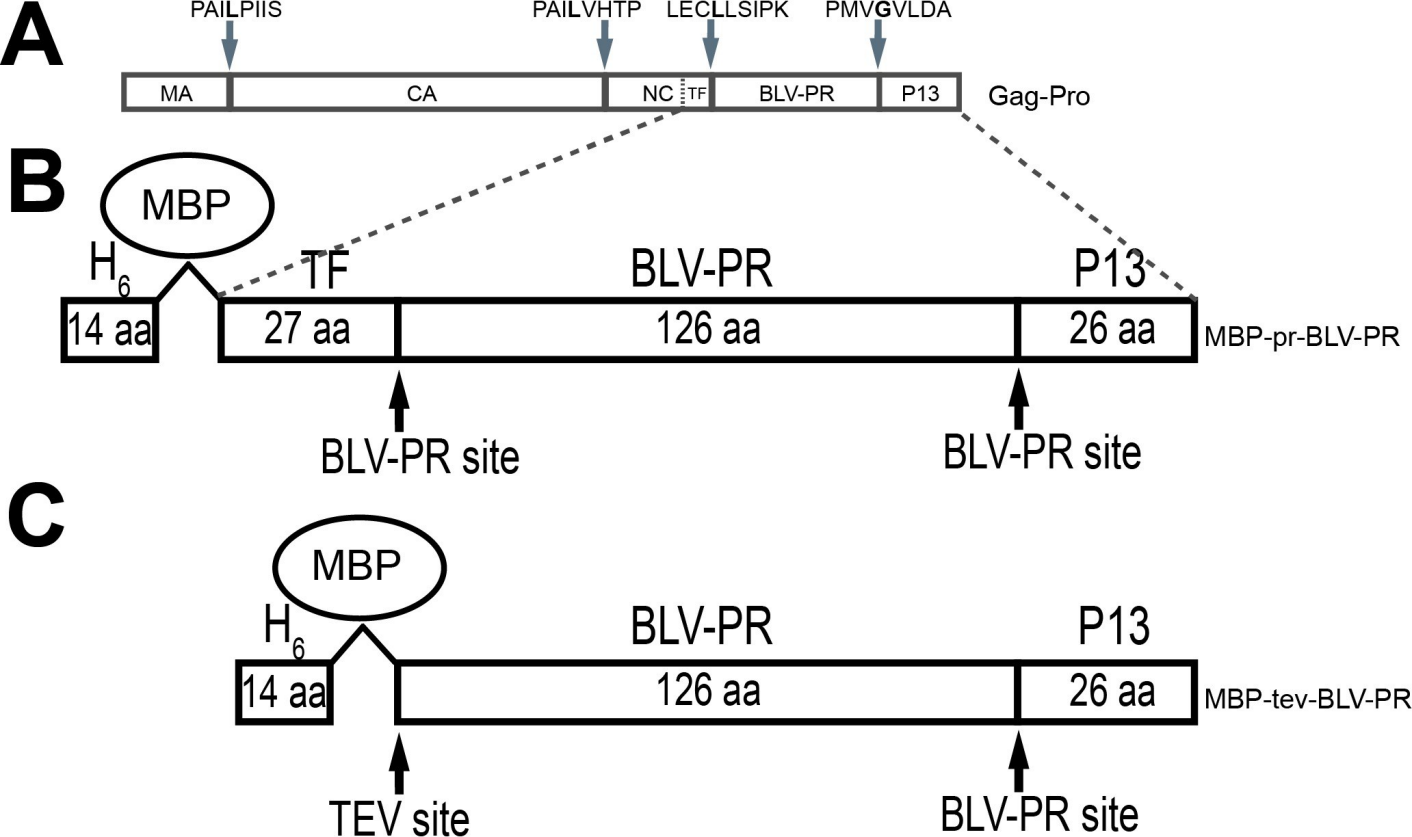
Constructs for expression of BLV-PR. (A) Schematic representation Gag-Pro, a precursor polyprotein of BLV. Gag-Pro is processed by BLV-PR, releasing matrix (MA), capsid (CA) and nucleocapsid (NC) proteins. Immature BLV-PR is fused to the C-terminal end of Gag, separated by a transframe region (TF), and with a peptide extension (P13) at the C-terminus. The hydrolysis sites for BLV-PR are indicated by grey arrows with the amino acid sequence. P1 is leucine for MA-CA, CA-NC and TF-PR while P1 is glycine for PR-P13. A part of the polyprotein is expanded in the panels below. (B) MBP-pr-BLV-PR. This BLV-PR expression construct contains TF and P13 of immature BLV-PR, with the native hydrolysis sites for BLV-PR. The N-terminal end is linked to maltose binding protein (MBP) and has a tandem of six histidines (H_6_) for affinity purification. (C) MBP-tev-BLV-PR. This construct contains MBP with H_6_, separated from BLV-PR by a hydrolysis site for TEV protease (ENLYFQL, with leucine in P1´). The black arrows show the hydrolysis sites.

It has been proposed that BLV targets bovine B lymphocytes through the binding of viral envelope glycoprotein (Env) to a cellular receptor [10]. Once infected host cells, the viral genome is retrotranscribed and integrated as a provirus into the genome of the host infected cell [11, 12]. After transcription to mRNA, viral proteins are translated and some unspliced copies of mRNA functions as new copies of the viral genome. The novel Gag proteins bind to copies of the viral RNA genome and package and exit the cell as non-infective virus particles. A crucial point in the replicative cycle of retroviruses is the successful transition from a non-infective virus particles to an infective virion, and the viral retropepsin (PR), a homodimeric aspartic protease, is the main actor involved in this process [13]. Given the biological importance of PR in the viral replication mechanism, the pharmacological use of specific inhibitors of proteolytic activity constitutes a therapeutic strategy with potential clinical impacts [14]. In fact, the PR of *Human immunodeficency virus* (HIV) is one of the main targets of antiretroviral drugs, and several inhibitors are currently used in the antiviral pharmacological treatment [13, 15, 16]. On the other hand, until now there are no direct-acting antivirals against HTLV targeting HTLV-PR, however, recent works demonstrate that there is active interest in the search and study of new HTLV-PR inhibitors [17, 18]. The main substrate of PR is Gag, a precursor polyprotein relevant for capsid self-assembling. Gag is processed by PR, releasing matrix (MA), capsid (CA) and nucleocapsid (NC) proteins, which undergo dramatic conformational rearrangments which confer infectivity to virions [19]. Other longer versions of Gag are also expressed and processed by PR. In BLV there are two such versions, one is Gag-Pro (Fig 1A), in which an immature PR is fused to the C-terminal end of Gag, separated by a transframe region (TF) and with a peptide extension (P13) at the C-terminus. The other one is Gag-Pro-Pol, which includes the other enzymes of the virus (reverse transcriptase and integrase), also released by PR [20–22].

Self-hydrolysis at specific sites releases the PR monomer, which then dimerizes to generate the active form of the enzyme [22, 23]. Since the N- and C-termini are important for dimerization [24], the correct self-processing appears to be relevant for activity. Active retroviral PRs are composed of two identical monomers of 10–15 kDa (14 kDa in BLV-PR). Each monomer contributes an Asp residue together with Thr/Ser (Thr in BLV) and Gly, that form only one catalytic triad per dimer [16, 22]. Near the active site, a network of hydrogen bonds provides relative rigidity to the enzyme core [25]. Two glycine-rich F062-hairpins, or flap tips, cover the active site cleft in a way reminiscent of a dynamic sunroof. These flexible regions play a very important role in substrate recognition, and undergo significant conformational changes between closed, semi-open and open topologies. The open conformation initiates the interaction with the substrate; after substrate binding, the flap changes to a closed state in which the substrate is processed. Then, the flap opens and the two products are released [16, 26–28]. The PR from different retroviruses recognizes different amino acid sequence along the polyprotein substrate, indicating enzymatic promiscuity [22]. To date, no experimental data on BLV-PR structure are available; in fact, experimental structural data regarding BLV is limited to CA (which forms a crystallographic hexagonal lattice resembling viral arrangements), as previously reported by our group [29], and a trimeric hairpin domain corresponding to the membrane proximal fragment of the Env glycoprotein [30]. However, a molecular model of BLV-PR was proposed from analysis of the sequence comparison of BLV-PR with the HIV-1-PR and HTLV-1-PR of known structures by Sperka et al [31]. Although they have approximately 20 and 30% primary sequence similarity to BLV-PR, respectively, this molecular model of BLV-PR was predicted to share the conserved core regions of PRs.

Protease activity is relevant in the viral maturation process that generates infective retrovirus particles. HIV-PR has been extensively studied; however there is scarce information on the kinetic parameters that characterize the enzymatic activity of BLV-PR. In previous works [31, 32], BLV-PR was expressed in *E*. *coli* as inclusion bodies, and the enzymatic activity was studied following the hydrolysis of four different peptide substrates by reverse phase HPLC. This experimental approach were pioneering, contributing to the BLV-PR characterization. However it has some limitations such as low analytical sensitivity impeding the proper estimation of K_M_ values lower than 10 μM as in the case of MA-CA, CA-NC and TF-PR substrates. Besides, this methodology does not allow analyzing time courses of proteolytic reactions.

In this work, we aimed to improve the characterization of the BLV-PR enzymatic activity. To do this, we generated a recombinant expression system in *E*. *coli* that enabled us to obtain BLV-PR in a soluble form with a high degree of purity. To measure the enzymatic activity of BLV-PR we designed four fluorogenic peptide substrates containing the hydrolysis sites TF-PR, PR-P13, MA-CA and CA-NC. Our steady-state and pre-steady-state kinetic studies using these substrates revealed that the enzyme dimerizes and undergoes rate-limiting conformational changes.

## Materials and methods

### Preparation of recombinant BLV-PR

BLV-PR was expressed in *E*. *coli* strain BL21(DE3) pLysS, fused to maltose binding protein with a six-histidine tag at its N-terminal end (H6-MBP) using a modified pQE80L recombinant plasmid [33]. The expression constructs included the sequence of H6-MBP followed by full-length BLV-PR (GenBank: AAB50411.1). To maintain the original leucine in the N-terminus of BLV-PR, two strategies were used. The first construct, MBP-pr-BLV-PR, coded for the self-activating protein and contained the BLV-PR sequence flanked by the TF (27 amino acids) and P13 (25 amino acids) extensions at the respective N- and C-terminal ends, with the BLV-PR hydrolysis sites resembling the immature wild type Gag-Pro polyprotein (Fig 1B). The second construct, MBP-tev-BLV-PR, had the N-terminal TF extension substituted by a tobacco etch virus (TEV) protease hydrolysis site with leucine in P1´; this second construct coded for a TEV protease-activatable BLV-PR (Fig 1C, protein sequences in S1 File).

The genes were synthesized after codon optimization for *E*. *coli* expression (Genscript, NJ, USA). The constructs included sequences of generic primers for restriction free (RF) cloning using Phusion DNA polymerase (New England Biolabs) in modified pQE80L vectors [33]. The sequence of the expression constructs was verified by automated sequencing (Macrogen).

Transformed bacteria were grown with agitation at 37°C in 2YT medium containing 100 μg/L ampicillin until late-log-phase (absorbance at 600 nm of 1.0 ± 0.2) and induced with 0.1 mM isopropyl thiogalactopyranoside (IPTG) for 4 h. Cells were harvested by centrifugation (4000 g, 30 min, 4°C), resuspended in lysis buffer (50 mM Tris, pH 8, 300 mM NaCl, 10 mM imidazole), and sonicated. The lysates were centrifuged (20,000 g, 30 min, 4°C) and the supernatants were loaded into a Ni-NTA affinity matrix column (Ni-NTA, Invitrogen) equilibrated with lysis buffer. The column was washed with the same buffer including 10 mM imidazole, and the His-tagged fusion protein was eluted with a gradient from 10 to 500 mM imidazole in the same buffer using an AKTA Purifier (Cytiva) FPLC system. In the case of MBP-tev-BLV-PR construct, the eluate was dialyzed together with His-tagged TEV protease (produced in-house) [34] (1 mg each 5 mg of recombinant protein) in 50 mM Tris, pH 8, 300 mM NaCl and 0.1 mM DTT, and then reinjected onto a Ni-NTA column to retain His-tagged TEV protease and unprocessed products, to obtain the mature pure protein in the flow-through fraction. The buffer was changed to 50 mM Tris, pH 8, 10 mM NaCl using a PD10 column (Sigma-Aldrich), and ion exchange chromatography was performed using a Mono S HR 5/50 column (GE Healthcare) equilibrated in the same buffer and eluted with a gradient of 10 mM to 500 mM NaCl. Finally, size exclusion chromatography was performed using a Superdex 75 100–300 analytical column (GE Healthcare) in 50 mM Tris buffer, pH 8.0, 100 mM NaCl at 1 mL/min, following absorbance at 280 nm.

Purified fractions were analyzed by 15% SDS-PAGE and mass spectrometry. The purity of BLV-PR used for activity studies was always > 95%. The concentration of the protein was estimated from the absorbance at 280 nm using sequence-based extinction coefficients calculated with the ProtParam tool available at ExPASy server (https://web.expasy.org/cgi-bin/protparam/protparam). Peptide mass fingerprinting of recombinant BLV-PR was performed by in-gel trypsin (Sequencing-grade, Promega) treatment of an SDS-PAGE band followed by matrix assisted laser desorption ionization-time of flight mass spectrometry (MALDI-TOF MS) of the tryptic digest (4800 MALDI TOF-TOF Analyzer System, Applied Biosystems). Peptides were extracted from the gel in 60% acetonitrile, 0.2% trifluoroacetic acid, concentrated by vacuum-drying and desalted using C18 reverse phase micro-columns (OMIX Pipette tips, Varian). The sequence of selected peptides was verified.

### Design of substrates for kinetic studies

Fluorogenic peptide substrates for BLV-PR, in which fluorescence is enhanced by peptide hydrolysis, were designed based on the Förster resonance energy transfer (FRET) between the donor 5-((2-aminoethyl)amino)naphthalene-1-sulfonic acid (EDANS) and the acceptor 4-((4-(dimethylamino)phenyl)azo)benzoic acid (DABCYL), as previously described for other proteases such as HIV-PR [35]. BLV-PR substrates consist of 12 amino acids, which contains one arginine at each ends to improve their solubility. The second amino acid is a glutamic acid modified with EDANS. The amino acid in position 11 is a lysine modified with DABCYL. The remaining amino acids correspond to specific hydrolysis sites, and contain the sequences corresponding to: a) the matrix-capsid hydrolysis site (MA-CA: RE(EDANS)PAILPIISK(DABCYL)R); b) the capsid-nucleocapsid hydrolysis site (CA-NC: RE(EDANS)PAILVHTPK(DABCYL)R); c) the hydrolysis site in the transframe N-terminal side of immature BLV-PR (TF-PR: RE(EDANS)LECLLSIPK(DABCYL)R); and d) the hydrolysis site in the C-terminal side of immature BLV-PR (PR-P13: RE(EDANS)PMVGVLDAK(DABCYL)R) (Fig 1A). Peptides were custom-made by United Biosystems.

### Activity assays

The activity of enzyme preparations was determined with the fluorogenic substrates at 37°C in 250 mM phosphate buffer, pH 5.6, 500 mM NaCl, 10 mM EDTA, 5 mM DTT, 1% glycerol, 1% DMSO (reaction buffer). This activity buffer was chosen to facilitate comparison with published data [31, 32]. The formation of the EDANS-containing hydrolysis product was registered at excitation and emission wavelengths of 340 and 490 nm, respectively. Assays were performed using either 200 μL samples in black 96-well microplates (Costar, Corning Life Sciences) read in a microplate fluorescence reader (FLUOstar* OPTIMA, BMG Labtechnologies), or in 110 μL sub-micro cells (Type 16F Starna Cells) read in a Varian Cary spectrofluorometer. Calibration curves using EDANS were carried out in each experiment, in the presence of the substrate concentration used for each reaction. The steady-state rates of substrate hydrolysis were calculated from the linear portion of product versus time plots corresponding to less than 10% of substrate consumption.

The values of *K*_M_ and *k*_Cat_ for each substrate were calculated from plots of steady-state rate versus substrate concentration (0.5–100 μM) in the presence of 10 nM BLV-PR. The steady-state rates were determined in the interval between 1 and 2 h after adding enzyme to the reaction mixtures. Non-linear Michaelis-Menten equations were fit to the data. The parameter *k*_*C*at_ was also calculated from the linear fit of plots of steady-state rate versus enzyme concentration (1–200 nM), in the presence of 5 μM substrate. The slope of the plots is *k*_*C*at_[S]/(*K*_M_ + [S]). Values were averaged from independent duplicate measurements ± the standard error (n ≥ 3).

### Dimer-monomer dissociation studies

To determine the dimer-monomer dissociation constant (*K*_DM_), the initial rate (*v*) of MA-CA substrate hydrolysis was measured during the first 3 min of reaction using 1 μM substrate and increasing concentrations of BLV-PR (5 to 200 nM, expressed as the concentration of monomer). The enzyme was previously incubated in reaction buffer for 15 min at 25°C to achieve dimer-monomer equilibrium. The dilution of the enzyme in the activity mixture upon addition of the substrate was negligible (only 1.1-fold). The *K*_DM_ values, were averaged from independent duplicate measurements ± the standard error (n ≥ 3), were calculated by nonlinear fitting of the quadratic Eq (1), where [E]_t_ represents the total enzyme concentration, expressed as monomer, and *A*_*s*_ is the specific activity of the dimer [36]:

$$v=\frac{A_{s}\left[K_{DM}+4{\left[E\right]}_{t}-\sqrt{K_{DM}^{2}+8K_{DM}{\left[E\right]}_{t}}\right]}{8}$$ (1)

### Time courses of substrate hydrolysis

Progress curves were followed using increasing enzyme and substrate concentrations. Reactions were started by diluting the enzyme stock 4- or 40-fold in the activity mixture. Time courses were analyzed using the exponential plus straight line Eq (2) that describes the slow establishment of steady state conditions between the enzyme and substrate:

$$[\text{Product}]=offset+Amp*e^{\left(-kobs*t\right)}+v_{ss}t$$ (2)

where *offset* is a constant, *Amp* is the amplitude of the exponential phase, *k*_*obs*_ is the observed exponential pseudo-first order rate constant and *v*_*ss*_ is the slope of the linear phase and represents the steady state rate of the reaction. Computer fitting of progress curves gave estimated values for these parameters.

## Results

### Expression and purification of BLV-PR in its soluble form and with the original leucine in its N-terminus

Two constructs of recombinant BLV-PR were expressed in *E*. *coli* (Fig 1). Both of them were fused to H6-MBP at the N-terminus and to the 26 amino acid extension corresponding to P13 of the immature protein, with the respective BLV-PR hydrolysis site, at the C-terminus. One of the constructs, named MBP-pr-BLV-PR, also had, between MBP and BLV-PR, the 27 amino acid extension corresponding to the TF region of the immature polyprotein, including the native hydrolysis site for BLV-PR self-activation. The other construct, MBP-tev-BLV-PR, did not include the TF region nor the native hydrolysis site for BLV-PR self-activation. Instead, it presented a hydrolysis site for TEV protease between MBP and BLV-PR, including a leucine in the P1´ residue in lieu of the corresponding glycine or serine residue. Although the efficiency of TEV protease hydrolysis may decrease by this modification, mature BLV-PR without additional amino acids could be prepared.

**Fig 2:**
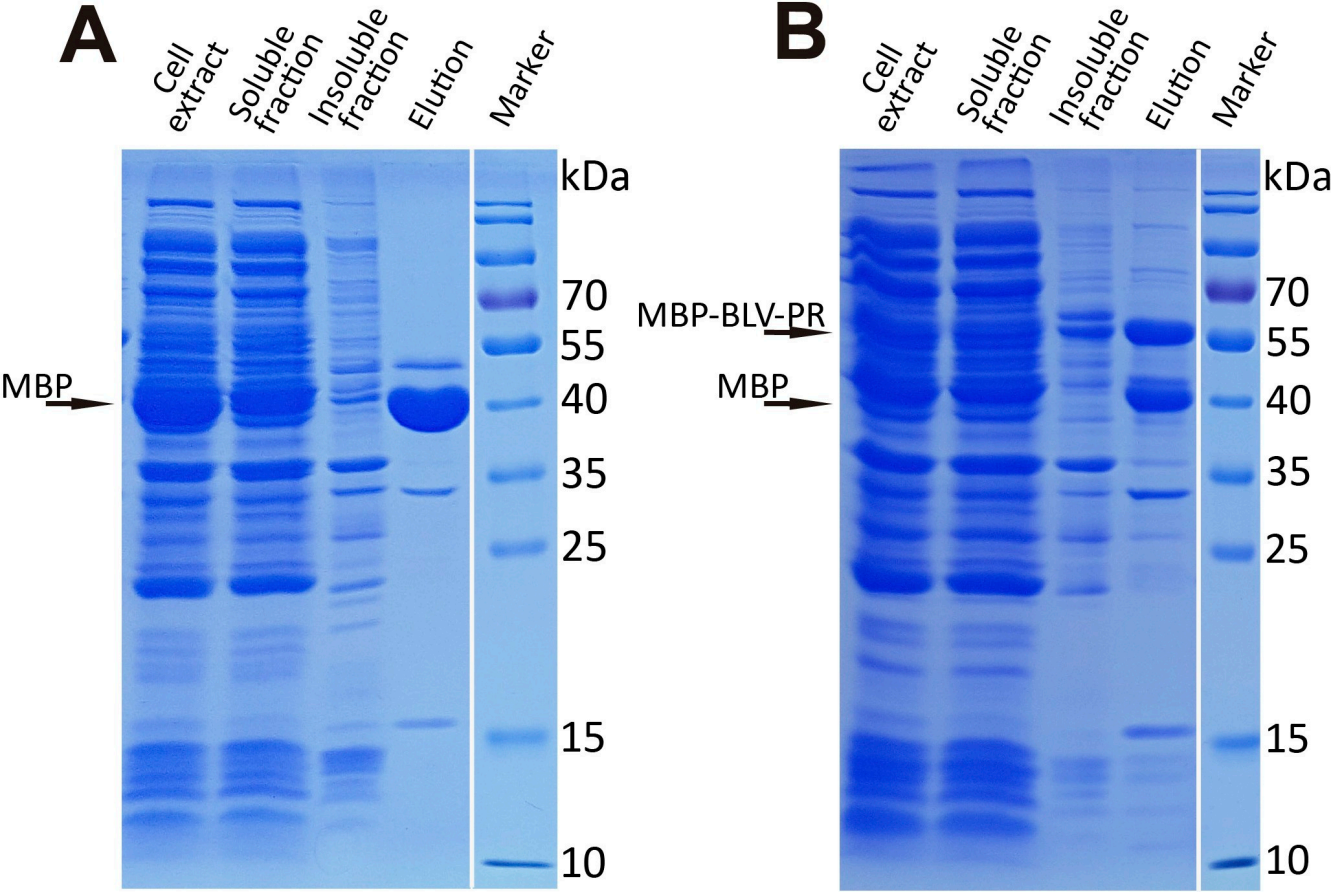
SDS-PAGE analysis of the expression of the PR of BLV constructs in *E*. *coli* and the purification step by nickel affinity chromatography. Expression of the construct MBP-pr-BLV-PR **(A),** and MBP-tev-BLV-PR **(B)**. The arrows indicate the bands corresponding to MBP and MBP-BLV-PR. The vertical white line indicates the removal of non-relevant lanes.

The different fractions obtained from the purification of the two BLV-PR constructs were analyzed by SDS-PAGE. In the case of MBP-pr-BLV-PR (Fig 2A) an intense band corresponding to MBP alone (42 kDa) was observed, and its identity was verified by mass spectrometry. In contrast, we did not detect bands corresponding to MPB-linked BLV-PR protein (63 kDa) nor mature BLV-PR (14 kDa), with similar intensity. In addition, another low intensity band of ⁓ 50 kDa was detected, and mass spectrometry analysis confirmed that it was composed of MBP fused to a fragment of the recombinant BLV-PR, as a product of partial maturation. Taken together, the absence of MBP-BLV-PR and BLV-PR, and the presence of two MBP containing proteins with different sizes indicated that in this construct BLV-PR was expressed, self-activated, and finally degraded within *E*. *coli*.

**Fig 3:**
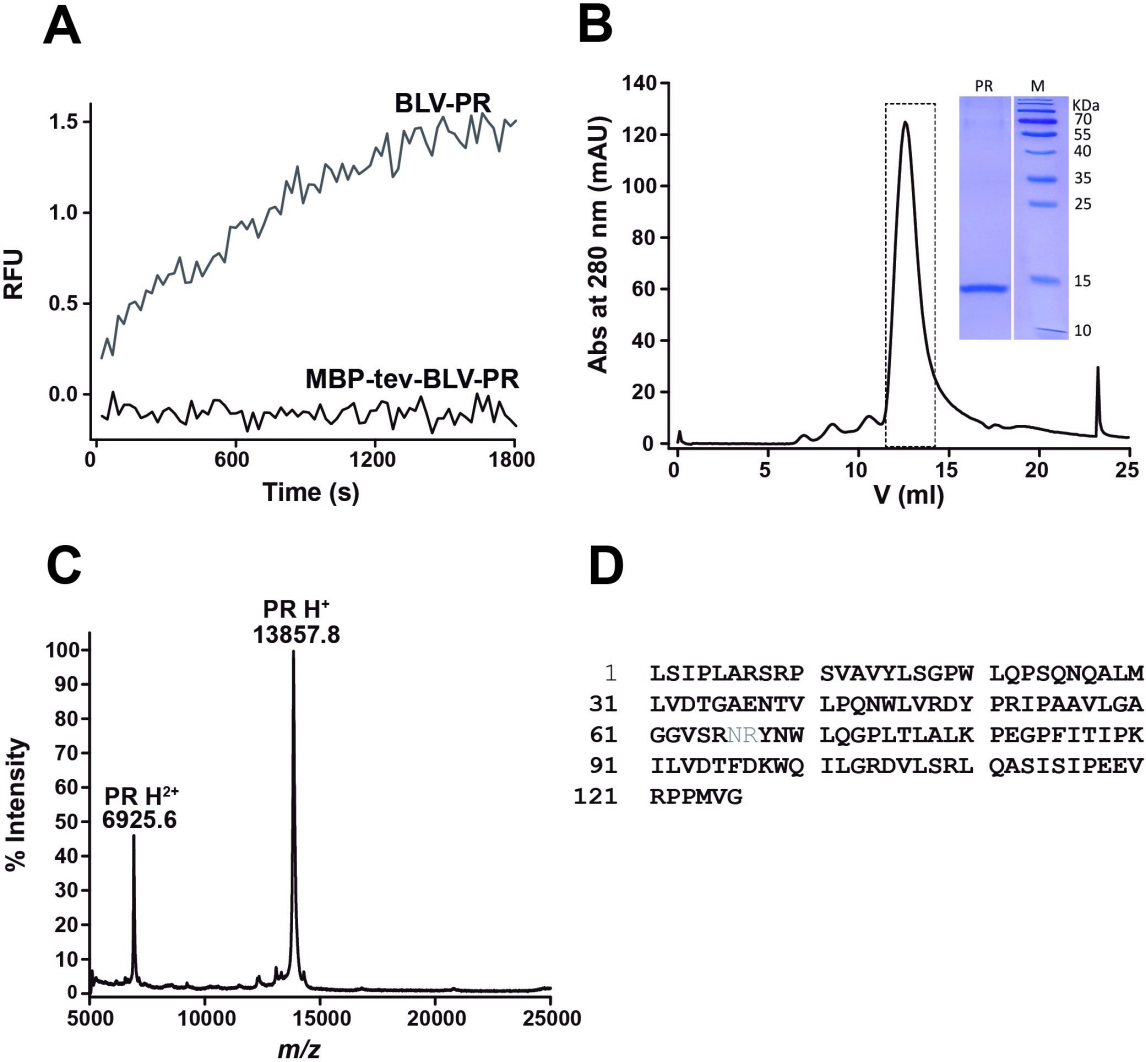
Activation and purification of mature BLV-PR. **(A).** Enzyme activity assay of the fractions eluted from the nickel affinity purification step, before and after treating with TEV (1mg each 5 mg of protein), MBP-tev-BLV-PR and BLV-PR respectively. The enzyme (1 μL of a 1:100 dilution of the eluted fraction) was added to reaction mixtures containing the fluorogenic substrate (MA-CA, 5 μM) in reaction buffer. **(B).** Final purification step by size exclusion chromatography using Superdex 75 10/300 column previously equilibrated with Tris (50 mM, pH 8.0, 100 mM NaCl). The inset shows the SDS-PAGE analysis of the fraction collected. **(C).** MALDI-TOF MS of purified BLV-PR. The peak at *m/z* 13857.8 Da matched the MH^+^ value predicted for BLV-PR (13871.5 Da). **(D)** Observed coverage (98%) of the peptide mapping of BLV-PR treated with trypsin by mass spectrometry, the identified residues are highlighted in black.

With the MBP-tev-BLV-PR construct, we successfully prevented BLV-PR maturation during the expression and purification steps. The SDS-PAGE analysis (Fig 2B) showed that the elution fraction of the Ni-NTA column contained two main proteins whose identity was confirmed by mass spectrometry. The ⁓42 kDa band corresponded to MBP, and the ⁓57 kDa band to MBP-tev-BLV-PR. The presence of MBP alone is characteristic of this expression vector, since it has been observed with several constructs [33]. According to the mass spectrometric analysis, the MBP-tev-BLV-PR protein was correctly hydrolyzed at the C-terminal BLV-PR site. Nevertheless, the enzymatic activity of MBP-tev-BLV-PR appeared to be low, given that no activity with fluorogenic substrates was observed, while activity was indeed observed after separating BLV-PR from MBP with TEV protease (Fig 3A). Once BLV-PR was activated by TEV protease treatment, ion exchange and size exclusion chromatography steps were performed to complete the purification. A single peak was observed by size exclusion chromatography (Fig 3B). The final yield obtained was 0.5–1 mg per liter of culture. The homogeneity and purity of the sample was verified by SDS-PAGE, and more than 95% protein purity was obtained. Mass spectrometry confirmed that BLV-PR matured correctly. A peak at *m/z* 13857.8 Da matched the MH+ value predicted for BLV-PR (13871.5 Da), the difference being within the measurement error of the equipment (Fig 3C). The analysis of the peptides obtained after treating BLV-PR with trypsin showed a coverage of 98%, and confirmed the correct processing of the N- and C-termini (Fig 3D).

Taken together, our results show that the strategy involving H6-MBP tag followed by a TEV protease hydrolysis site allowed us to obtain soluble and mature recombinant BLV-PR, in relatively good yields, and with the same amino acid composition as the native protein.

### The use of fluorogenic peptide substrates provide kinetic evidence for the existence of two conformational states

We tested the enzymatic activity of BLV-PR with fluorogenic peptide substrates containing the sequences corresponding to the hydrolysis sites between MA and CA (MA-CA), CA and NC (CA-NC), TF and BLV-PR (TF-PR) and BLV-PR and P13 (PR-P13). Assays were performed using either 200 μL samples in black 96-well microplates or in 110 μL sub-micro cells. Both assays yielded similar results. Preliminary experiments showed that all four substrates could be hydrolyzed by BLV-PR (S1 Fig in S1 File).

**Fig 4:**
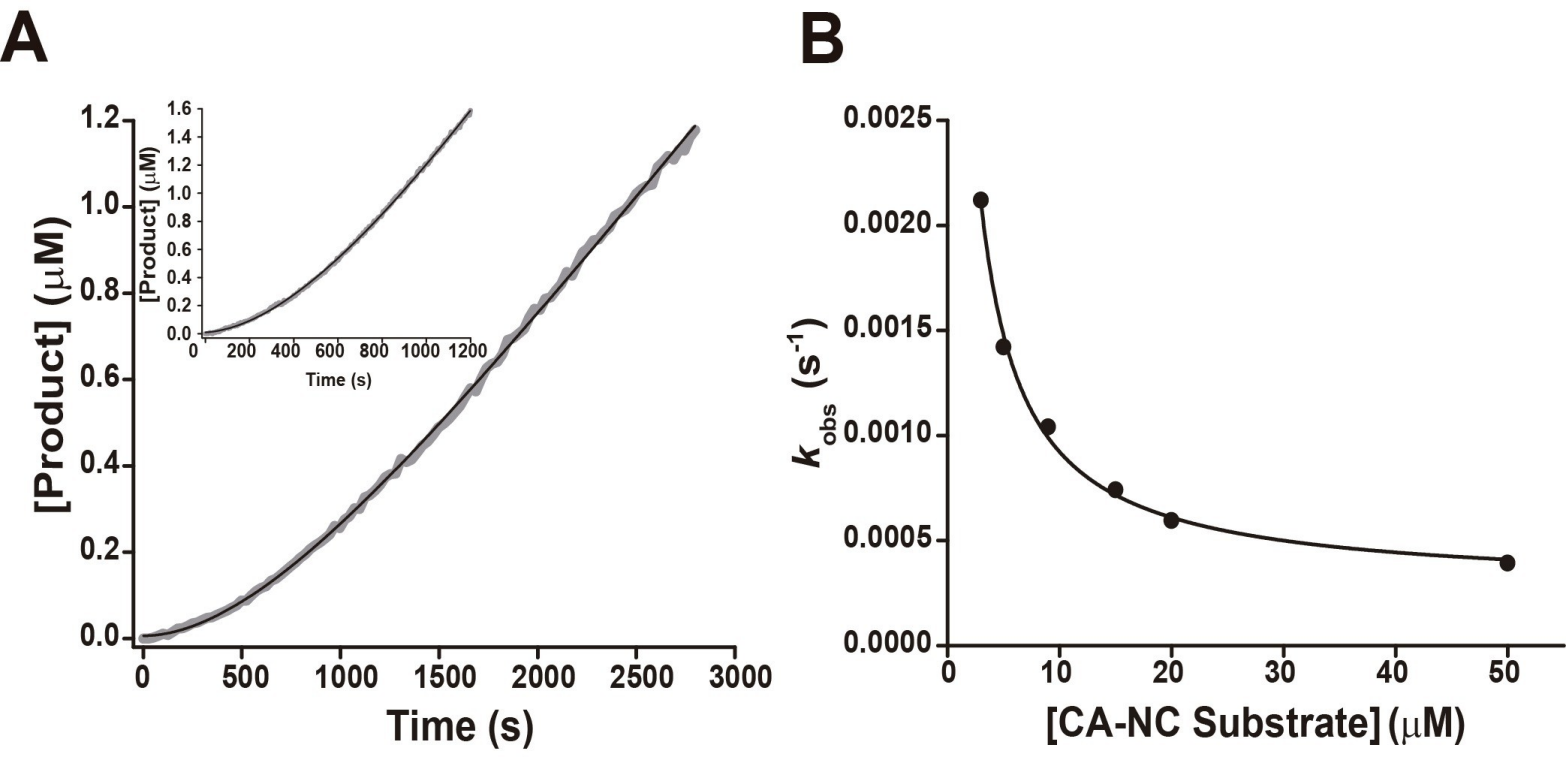
Kinetics of product formation of BLV-PR. **(A)** Progress curves for a representative experiment. The enzyme (5 nM, final concentration after 4-fold dilution) was added to reaction mixtures containing CA-NC substrate (5 μM) in buffer reaction, at 37°C. The black trace represents the best fit of Eq 2, from which the exponential rate constant *k*_obs_ was obtained. The inset shows the progress curve when 5 μl of concentrated PR (2.5 μM) were added to 195 μl reaction mixtures containing the CA-NC substrate (20 μM) in the same buffer as in (A). **(B)** Dependence of *k*_obs_ on the concentration of the CA-NC substrate (1–50 μM). The data points represent measurements in a representative experiment. The black trace represents the best fit of Eq 3 in agreement with Scheme 2.

When reactions were started with enzyme, by diluting the enzyme 4–fold in the final reaction mixture, the time courses of product formation had an upward curvature, indicating that the enzymatic activity increased as a function of time until it reached a steady state (Fig 4A). This behavior was observed with all four substrates. Our first hypothesis for this increase in activity was the slow establishment of an equilibrium between monomer (M) and dimer (E) where only the dimeric enzyme was able to react with the substrate (Scheme 1).


$$\text{M}+\text{M}\overset{k_{-\text{DM}}}{\underset{k_{\text{DM}}}{⇄}}\text{E}\overset{k_{\text{on}}[\text{S}]}{\underset{k_{\text{off}}}{⇄}}\text{ES}\overset{k_{\text{Cat}}}{\to }\text{E}+\text{P}$$ Scheme 1

However, when the reaction was started by adding 5 μl of concentrated BLV-PR (2.5 μM, ~ 30 times the *K*_DM_ value, see below) to 195 μl of the CA-NC substrate (20 μM, 5 times above the *K*_M_ value, see below) the time course showed the same behavior with an upward curvature (Fig 4A). Assuming that 2.5 μM BLV-PR is mostly in its dimeric form at the start of the reaction, the time-dependent increase in activity cannot then be exclusively explained by dimerization.

The time course kinetic studies were deepened using the CA-NC substrate. Exponential plus straight line equations (Eq 2) fitted the time course data very well (Fig 4A). From the fits to this equation, the apparent pseudo-first order exponential rate constants *k*_obs_ were determined. Remarkably, *k*_obs_ values decreased as the substrate concentration was increased (Fig 4B). This result is diagnostic, in the framework of a conformational selection model [37], of the existence of two enzyme conformations that interconvert relatively slowly, one (E*) that does not interact with the substrate and one (E) that does (Scheme 2). The conformational change between E* and E is the main cause of the time it takes for the system to reach the steady state.


$$\text{E}*\overset{k_{\text{r,app}}}{\underset{k_{\text{-r}}}{⇄}}\text{E}\overset{k_{\text{on}}[S]}{\underset{k_{\text{off}}}{⇄}}\text{ES}\overset{k_{\text{Cat}}}{\to }\text{E}+\text{P}$$ Scheme 2

For this reaction scheme, if the substrate binding and dissociation steps (*k*_on_[S] and *k*_off_, respectively) are fast compared to the conformational change between E* and E (*k*_r,app_ and *k*_-r_), the exponential rate constant *k*_obs_ shows an inverse hyperbolic dependence with the substrate concentration, and is related to the apparent kinetic constants of the conformational change, *k*_r,app_ and *k*_-r_, and to the equilibrium dissociation constant of the substrate, *K*_S_, defined as *k*_off_/*k*_on_, according to Eq 3 [37]:

$$k_{obs}=k_{r,app}+k_{-r}\left(\frac{K_{S}}{K_{S}+[S]}\right)$$ (3)

From the fit of Eq 3 to the *k*_obs_ vs [S] data plot (Fig 4B), the apparent kinetic constants involved in the conformational change were determined to be: *k*_r,app_, 2.9 ± 0.2 X 10^−4^ s^-1^; *k*_-r_, 9.5 ± 3.0 X 10^−3^ s^-1^; and *K*_S_, 0.8 ± 0.4 μM. These values are the averages of independent measurements ± the standard error (n = 4). The *k*_r,app_ value probably represents an apparent rate constant, affected by dimer-monomer dissociation. It is likely that the conformational change that was evidenced kinetically is in fact the transition from the closed to the open state of the active site flap, which has been observed in structural studies of HIV-PR [26–28, 38, 39]. The values of the rate constants suggest that, in the absence of substrate, the predominant form of BLV-PR is the closed state.

### Determination of the kinetic parameters for the four fluorogenic peptide substrates

**Fig 5:**
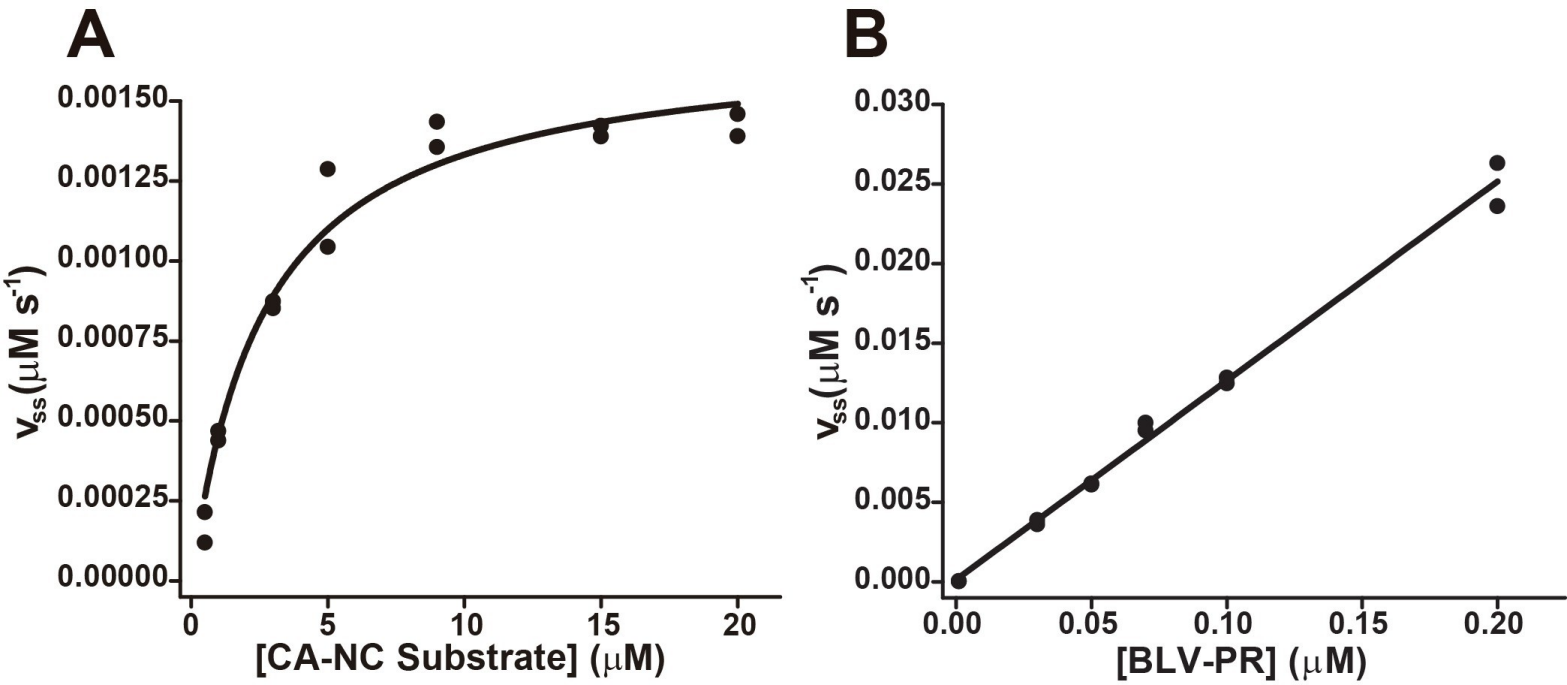
BLV-PR Kinetic parameters determined by using fluorogenic substrates. **(A)** Steady-state rate as a function of the CA-NC substrate concentration. BLV-PR (10 nM) was mixed with substrate CA-NC (0.05–20 μM) in reaction buffer, at 37°C, and the steady-state rate of hydrolysis was determined from the linear portion of the fluorescence increase. The black trace represents the best fit of the Michaelis-Menten equation. **(B)** Steady-state rate as a function of the enzyme concentration. CA-NC substrate (5 μM) was mixed with BLV-PR (0.05–0.2 nM) in the same buffer. The black trace represents the best fit to a linear equation. The data points represent independent steady-state rate measurements for a representative experiment performed in duplicates.

The kinetic parameters for each substrate were determined from measurements of the steady state rate as a function of substrate concentration. The values of *K*_M_ were in the micromolar range (Fig 5A and Table 1). Importantly, the value of *K*_M_ for the substrate CA-NC (3.4 ± 0.2 μM) was reasonably consistent with the value of *K*_S_ calculated from the time course experiments (0.8 ± 0.4 μM), especially considering that the *K*_M_ value does not necessarily represent the equilibrium dissociation constant. From the V_Max_ values, after dividing by the total enzyme concentration (expressed as dimer), we obtained *k*_*C*at_ values. In addition, the *k*_*C*at_ values were also calculated from plots of steady state rate as a function of enzyme concentration obtained at a fixed substrate concentration of 5 μM, after correcting by the factor (K_M_ + [S])/[S]); the values obtained in this way were similar to those calculated from V_Max_ (Fig 5B and Table 1). Importantly, a linear behavior was observed in the steady state rate versus enzyme concentration plots, which suggests that, at the used substrate concentration, all enzyme was in the dimeric state. The specificity constant (*k*_Cat_/*K*_M_) was higher for the substrates CA-NC (60 ± 11 mM^-1^ s^-1^) and MA-CA (57 ± 11 mM^-1^ s^-1^), followed by TF-PR (18 ± 4 mM^-1^ s^-1^) and PR-P13 (1.0 ± 0.6 mM^-1^ s^-1^).

**Table 1:** Kinetic parameters of BLV-PR with the fluorogenic substrates.

	Substrate			
**parameter**	**MA-CA**	**CA-NC**	**TF-PR**	**PR-P13**
***K***_**M**_ **(μM)**^**(**^^**a**^^**)**^	**2.1 ± 0.3**	**3.4 ± 0.7**	**2.6 ± 0.2**	**10 ± 6**
**k**_**Cat**_ **(s**^**-1**^**)**^**(**^^**b**^^**,**^^**c**^^**,**^^**d**^^**)**^	**0.13 ± 0.03** ^**(c)**^	**0.33 ± 0.07** ^**(c)**^	**0.07 ± 0.01** ^**(c)**^	**0.012 ± 0.008** ^**(c)**^
	**0.12 ± 0.01** ^**(d)**^	**0.21 ± 0.05** ^**(d)**^	**0.05 ± 0.01** ^**(d)**^	**0.010 ± 0.002** ^**(d)**^
**k**_**Cat**_**/K**_**M**_ **(mM**^**-1**^ **s**^**-1**^**)**^**(e)**^	**57 ± 10**	**61 ± 11**	**18 ± 4**	**1.0 ± 0.6**

^(a)^ K_M_, the Michaelis-Menten constants, were calculated from the non-linear fits of *v* versus substrate concentration plots as in Fig 5A. Values are the averages of independent duplicate measurements ± the standard error (n ≥ 3).

^(b)^ k_Cat_, the catalytic constants, were calculated by two methods. Values are the averages of independent duplicate measurements ± the standard error (n ≥ 3).

^(c)^ k_Cat_ values were calculated from the non-linear fits of *v* versus substrate concentration plots as in Fig 5A.

^(d)^ k_Cat_ values were calculated from the linear fits of v versus enzyme concentration plots as in Fig 5B.

^(e)^ k_Cat_/K_M_, the specificity constants, were calculated from k_Cat_ values in ^(c)^ and K_M_ values.

### Estimation of the equilibrium constant of the dimer-monomer dissociation

**Fig 6:**
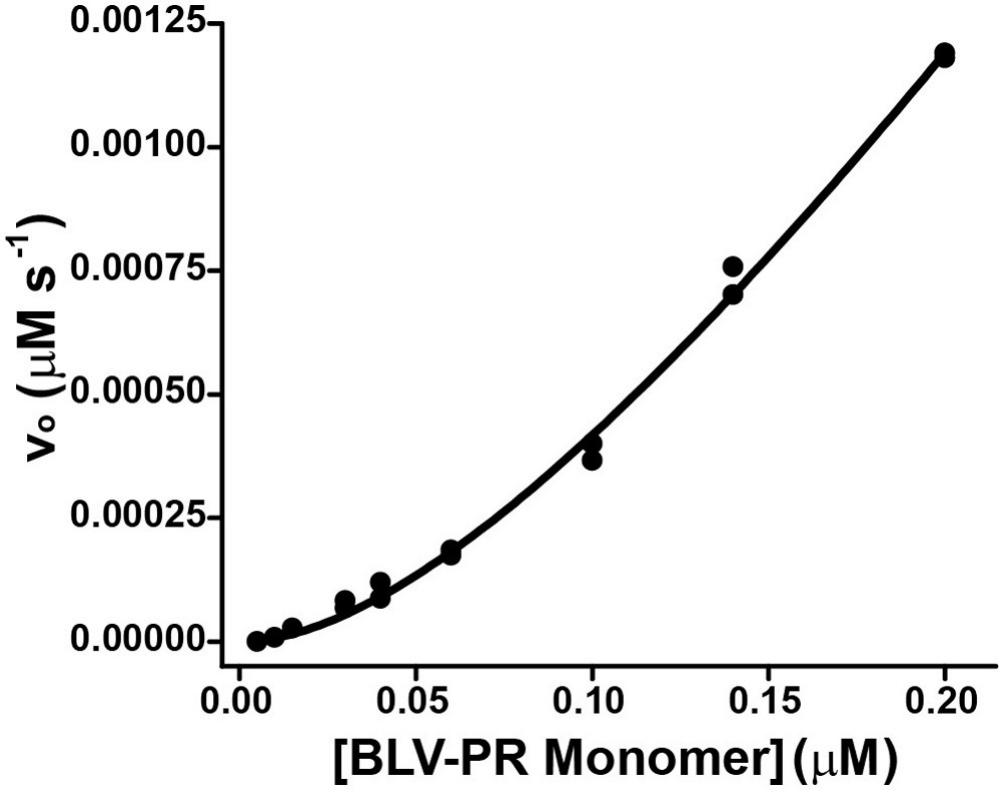
Initial rate as a function of enzyme concentration. The enzyme BLV-PR (0.005–0.2 μM, expressed as monomer) was preincubated for 15 minutes at room temperature and mixed with substrate (MA-CA, 1 μM), with minimal dilution (1.1-fold), in reaction buffer at 37°C. The initial rate of the reaction (v_0_) was determined form the first 3 min of the time courses of fluorescence increase. The data points represent independent measurements for a representative experiment performed in duplicates. The black trace represents the best fit of Eq 1, from which the equilibrium constant for dimer dissociation (*K*_DM_) was obtained.

To study the equilibrium between dimer and monomer, the initial rate during the first three minutes of the reaction was measured at increasing concentrations of total BLV-PR. It was assumed that in the first few minutes of the reaction, the activity corresponds to the proportion of enzyme that is in the dimeric form and in the open conformation in the absence of the substrate. The different enzyme dilutions were incubated for 15 minutes at 25°C to allow for dimer-monomer equilibration. Then, the reactions were started by adding the lowest possible MA-CA substrate concentration (1 μM). The substrate concentration was chosen to be lower than *K*_M_ (2.1 ± 0.3 μM for MA-CA) to minimize possible effects of the substrate on BLV-PR dimerization. In this experimental design, the enzyme was diluted only 1.1-fold upon addition of substrate. As predicted, plots of initial rate as a function of total enzyme concentration (expressed as monomer) did not present a linear behavior. In contrast, the plots were quadratic, evidencing the equilibrium between the monomer and the BLV-PR dimer. From the fit of Eq 1 to the experimental data the value of the dimer-monomer equilibrium dissociation constant (*K*_DM_) was determined to be 160 ± 30 nM (Fig 6).

### Numerical fittings of the time course data

Putting together the evidence for the existence of a conformational change in BLV-PR and the evidence for dimerization, Scheme 3 can be proposed.


$$\text{M}+\text{M}\overset{k_{-\text{DM}}}{\underset{k_{\text{DM}}}{⇄}}\text{E}*\overset{k_{\text{r}}}{\underset{k_{-\text{r}}}{⇄}}\text{E}\overset{k_{\text{on}}[\text{S}]}{\underset{k_{\text{off}}}{⇄}}\text{ES}\overset{k_{\text{Cat}}}{\to }\text{E}+\text{P}$$ Scheme 3

**Fig 7:**
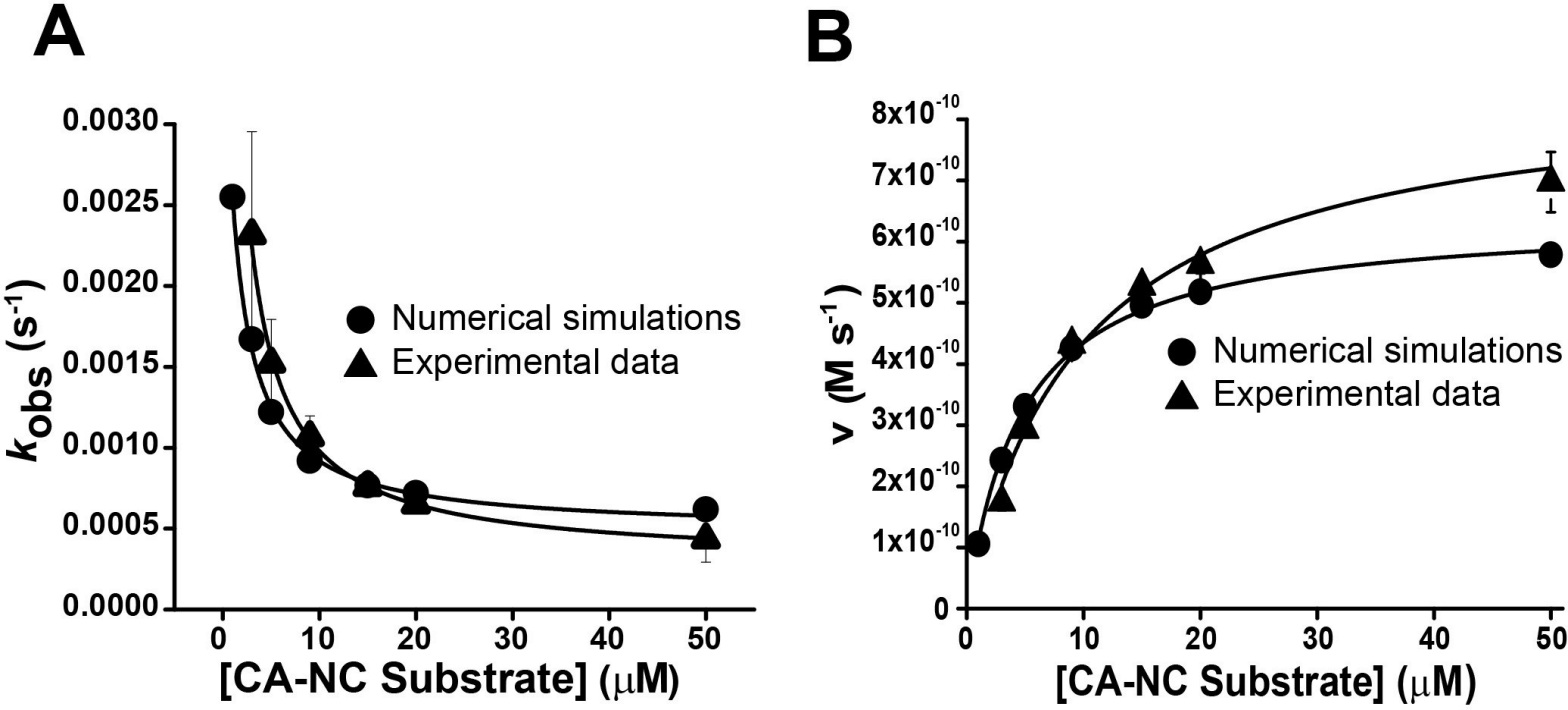
Confirmation of the plausibility of Scheme 3 through comparison between experimental and simulated data. **(A)** Dependence of experimental (triangles) and simulated (circles) *k*_obs_ on the concentration of the CA-NC substrate. The experimental values were obtained as in Fig 4 and represent the mean ± standard error (n = 4). The simulated data were obtained from numerical simulations using Scheme 3 and the rate constants obtained from time course experiments. The black traces represents the best fit of Eq 3. **(B)** Dependence of the experimental (triangles) and simulated (circles) steady state rate *v* on the concentration of the CA-NC substrate. The experimental values were obtained as in Fig 5 and represent the mean ± standard error (n = 4). The simulated data were obtained from numerical simulations as in (A). The black traces represents the best fit of the Michaelis-Menten equation.

In this reaction sequence, there is an equilibrium between the monomer (M) and dimer, the dimer alternates between a conformation that does not interact with the substrate (E*) and one that does (E), and the enzyme-substrate complex evolves to products, releasing E. To assess the feasibility of our proposal, we performed numerical fittings of this reaction sequence to several independent time courses at different substrate (CA-NC) and enzyme concentrations, using the COPASI software. Consistent with the obtained value of *K*_DM_ (160 nM), we arbitrarily set a value for the kinetic constant of monomer association (*k*_*-DM*_) of 95000 M^-1^ s^-1^, and a value for the kinetic constant of dimer dissociation (*k*_*DM*_) of 0.0152 s^-1^. The values of the other rate constants involved in the process were estimated with the numerical fittings. We obtained the following values: k_r_, 0.0029 ± 0.0009 s^-1^; k_-r_, 0.0052 ± 0.0007 s^-1^; k_on_ (21 ± 3) x 10^4^ M^-1^ s^-1^; *k*_off_ 0.00045 ± 0.00001 s^-1^ and *k*_Cat_ 0.10 ± 0.01 s^-1^. There was agreement between the value estimated in the numerical simulations for *k*_Cat_ and that obtained experimentally (Table 1). Besides, the values estimated from the fits of Scheme 3 to experimental data were used to make numerical simulations of time courses and fit the corresponding equations to them. These simulations were able to reproduce the behavior observed experimentally. In Fig 7 we shown that the shape of the experimental curve is similar to the theoretical one. The simulated *k*_obs_ values showed an inverse hyperbolic dependence with the substrate concentration, and fits of Eq 3 to simulated *k*_obs_ versus [S] data gave apparent values for *k*_r,app_ (4.9 ± 0.4 X 10^−4^ s^-1^), *k*_r_ (3.6 ± 3.0 X 10^−3^ s^-1^) and K_S_ (1.4 ± 0.2 μM), of the same order as those determined experimentally. In addition, fits of the Michaelis Menten equation to simulated steady state rate v versus [S] data gave values of *K*_M_ and *k*_Cat_ that were 4 μM and 0.1 s^-1^, respectively, consistent with those obtained experimentally. In the numerical fittings, more than one set of rate constant values could provide good fittings, depending on the values assigned to *k*_-DM_ y *k*_DM_. Nevertheless, the numerical simulation results support the feasibility of the reaction sequence proposed in Scheme 3, and that this scheme can explain the results obtained.

## Discussion

With the aim of expressing soluble recombinant BLV-PR in *E*. *coli* with good yield and with an intact N-terminus, two constructs of BLV-PR linked to H6-MBP were assessed, one in which BLV-PR matures by self-hydrolysis (MBP-pr-BLV-PR) and another one that requires treatment with TEV protease (MBP-tev-BLV-PR). With the MBP-pr-BLV-PR construct, neither MBP-BLV-PR nor mature BLV-PR were detected by SDS-PAGE. However, MBP was abundant in the soluble fraction, suggesting that the expression of the fusion protein was high, but that the protein matured and was degraded within *E*. *coli*. Results consistent with this were observed in the work of Zahuczky *et al*., in which mature BLV-PR was identified by western blot in the insoluble fraction, from which it was purified and then renatured [32]. On the other hand, when the MBP-tev-BLV-PR construct was expressed, an intense MBP-BLV-PR band was observed. This set of results suggests that both MBP-coupled BLV-PR constructs are expressed, but when the BLV-PR contains the BLV-PR site at the N-terminus, it matures within *E*. *coli* and is probably degraded, leaving only a low amount of protein in inclusion bodies, unobservable by SDS-PAGE. The instability of BLV-PR in *E*. *coli* can be a consequence of self-hydrolysis, as occurs with HIV and HTLV proteases [40, 41]. In fact, 5 μM BLV-PR (approximately sixty times the *K*_DM_), was almost completely degraded after 2 h incubation in the reaction buffer (S2 Fig in S1 File). In addition, the instability in *E*. *coli* may also be due to digestion by the bacterial systems, since the presence of a leucine residue at position 1 of the mature BLV-PR can function as a digestion signal in *E*. *coli* [42]. In this regard, the estimated half-life of BLV-PR in *E*. *coli* according to Expasy’s ProtParam software (https://web.expasy.org/cgi-bin/protparam/protparam) is 2 min, while the half-life predicted for the immature MBP-BLV-PR form is more than 10 h. Therefore, to express BLV-PR in *E*. *coli* in a soluble and active form, H6-MBP was linked at its N-terminus to improve the expression and facilitate the purification, and the native hydrolysis site between MBP and BLV-PR was eliminated. To activate the enzyme, a hydrolysis site for TEV protease instead of the native proteolytic site was included in the construct. This site contains leucine instead of the typical glycine in P1´. The inclusion of leucine in this position decreases the efficiency of hydrolysis by TEV, which can be compensated by increasing the concentration of the enzyme [43]. This strategy allows getting the expression of the mature BLV-PR including all its 126 amino acids and avoiding the presence of extensions at the N- and C-termini of the enzyme. This aspect is important since it has been observed in several viral homodimeric proteases that the inclusion of amino acid sequences at the N- and C-terminal ends can significantly affect enzymatic activity [44–46]. In addition, the inclusion of the TEV protease site in our expression system allowed us to obtain the native BLV-PR, preventing premature self-activation that leads to its degradation.

Several retroviral PRs have been expressed as MBP-fused proteins. Interestingly, the different proteins show variable solubilities and enzymatic activities, which suggests that the purification protocol needs to be adjusted in each case. For example, the MBP-PR from HIV goes to inclusion bodies or forms insoluble aggregates, and requires renaturation [47]. In addition, this construction had markedly decreased activity [47]. In contrast, the MBP-PR of *Human foamy virus* (HFV) presented activity when linked to MBP, and the hydrolysis site could be changed to that of factor Xa even though two extra amino acids were present at the N-terminus of the PR [48]. It is possible that the presence or absence of activity in PRs with extensions at the N-terminal end is a consequence of how these extensions affect dimerization in each case. The MBP-PR of HTLV, is released from MBP during expression and goes to inclusion bodies. It is not degraded within *E*. *coli*, and this is probably due to the fact that it has a proline in the N-terminal end which is not a function as a digestion signal in *E*. *coli* [42]. In addition, recombinant HTLV-PR had leucine 40 replaced by isoleucine. Leucine 40 is important for self-digestion [47, 49]. The protocol that we developed for BLV-PR, in which a hydrolysis site for another protease is incorporated, may be useful also to obtain soluble HTLV-PR.

We characterized the activity of BLV-PR with four FRET-based fluorogenic peptide substrates. The highest specificity constant was observed for the two hydrolysis sites that release capsid proteins, MA-CA and CA-NC (*k*_Cat_/*K*_M_ ~ 60 mM^-1^ s^-1^), followed by TF-PR (*k*_Cat_/*K*_M_ ~ 18 mM^-1^ s^-1^) and PR-P13 (*k*_Cat_/*K*_M_ ~ 1 mM^-1^ s^-1^) (Table 1). With these data, it is difficult to assign them a biologically relevant cleavage preference, since we ignore the effect of the context surrounding the hydrolysis cleavage sites in the polyprotein and the conditions of our assay do not necessarily reflect the environment within the virus particle. However, our data are comparable with those obtained by Zahuczky et al. and Sperka et al. [31, 32] who characterized the protease activity on peptide substrates with the same hydrolysis sites by using reverse phase chromatography (RP-HPLC) as analytical method. They observed the highest specificity constant for the substrate MA-CA (217 mM^-1^ s^-1^) followed by TF-PR (146 mM^-1^ s^-1^). These values are four and eight times higher, respectively, than those obtained with the fluorogenic substrates in this work. In the case of CA-NC, they reported a value of 63 mM^-1^ s^-1^ similar to the value that we calculated. Finally, for PR-P13 the *k*_Cat_/*K*_M_ was 0.4 mM^-1^ s^-1^, half that of the value obtained by using our fluorogenic peptide. Besides, the K_M_ values calculated for the fluorogenic substrates are consistent with those reported with peptide substrates, a K_M_ of approximately ⁓ 23 μM was reported for PR-P13 (twice that of the fluorogenic analogue). For the other three substrates, the reported *K*_M_ values are less than 10 μM, similar to those obtained herein with the fluorogenic substrates (see Table 1). In addition, very similar results were obtained for *k*_Cat_ with both fluorogenic substrates and the peptides. The k_Cat_ of the TF-PR substrate was not reported prior to this work. Overall, the kinetic parameters of both fluorogenic and unmodified peptides were similar. The highest differences were found in *k*_Cat_/*K*_M_, and can be explained by how they were calculated in both studies. In the case of the fluorogenic substrates, the *k*_Cat_/*K*_M_ was calculated by directly measuring *k*_Cat_ and *K*_M_, whereas in the case of the unmodified peptide substrates calculations involved competition experiments with substrates that present *K*_M_ values less than 10 μM, since the HPLC technique was not sensitive enough for direct calculations [31, 32]. The fluorophore moieties incorporated into the peptides do not appear to affect the hydrolysis significantly, allowing the characterization of the enzymatic activity. Therefore, as with other viral and non-viral protease substrates, the coupling of the peptide hydrolysis sites with fluorophores constitutes an excellent alternative for BLV-PR activity studies.

Through carefully designed activity measurements using the fluorogenic substrates, we studied the dimerization of BLV-PR. The dimer to monomer dissociation constant (*K*_DM_) that we determined is within the range of those calculated for other PRs [50]. However, it must be taken into account that the estimated *K*_DM_ values of viral PR can be markedly different depending on the experimental approach. For example, for the HIV-PR the *K*_DM_ values calculated with substrate-independent techniques, such as analytical ultracentrifugation, were in the order of 10^−5^ M, while in substrate-dependent studies, estimations showed a wide range, between 10^−7^ and 10^−9^ M [50]. Although there are differences in the experimental conditions between different studies, this important discrepancy in the values of *K*_DM_ is difficult to explain and makes it difficult to compare data. Nevertheless, the *K*_DM_ value that we obtained for BLV-PR is of the same order of magnitude (10^−7^ M) as that calculated by kinetic methods for the related HTLV-PR [51].

The time courses of substrate hydrolysis presented an exponential phase in which the rate increased with time before achieving a steady-state phase. The *k*_obs_ values decreased hyperbolically with substrate concentration, indicating that the time required to reach the steady state is not related (or not only) to dimerization, but rather, to a BLV-PR conformational change that modulates the interaction of the enzyme with the substrate. The inverse hyperbolic dependence on substrate concentration is diagnostic of a conformational selection model in which the enzyme is present in two conformational states that interconvert relatively slowly, and only one of which can interact with the substrate [37]. These conformational states could be a consequence of the opening and closing movement of the flaps which cover the enzyme’s active site. In this regard, HIV-PR in solution can adopt three different conformations in absence of the ligand: closed, semi-open and open, as a consequence of the flap movements. The less abundant conformational state is the open one, the state that can interact with the substrate [26–28, 38, 39]. In fact, HIV-PR NMR studies showed that the substrate protein domains that flank the hydrolysis site participate in the opening of the HIV-PR, and establish interactions that are important to determine the order in which viral polyproteins are hydrolyzed [52].

To sum up, we designed a new experimental strategy that allows expressing the mature recombinant BLV-PR in *E*. *coli*, obtaining the enzyme in its soluble form, with good yield and purity, and with native N- and C-termini. We characterized the enzymatic activities with four fluorogenic peptide substrates containing the specific hydrolysis site sequences for BLV-PR. The use of fluorogenic substrates opened the way to perform kinetic studies, which revealed the existence of dimerization and conformational change (probably corresponds to opening of the flaps covering the active site) steps. Both are important modulation points of the enzyme activity and therefore of the virus replicative cycle. The fluorogenic assay, in addition to being a very good strategy for kinetic studies of BLV-PR, can become a very useful tool for the search, characterization and design of inhibitory molecules that can modulate the different stages involved in the enzymatic activity such as the formation of dimers, the opening of the active site, or the catalytic mechanism itself. Obtaining molecules that efficiently inhibit BLV-PR could initiate the development of new pharmacological strategies aimed at controlling bovine leukosis, an infectious disease that has a very important impact on the productivity of dairy cattle. On the other hand, the study of BLV-PR may also be useful to improve the general understanding of other retroviral proteases such as those of HIV and HTLV.

## Supporting information

S1 Raw image (TIF)

S2 Raw image (TIF)

S1 File (ZIP)
