# Supplementary figures and images for: Kinetics of Bovine leukemia virus aspartic protease reveals its dimerization and conformational change
Source: PLoS One. 2022 Jul 22;17(7):e0271671. doi: 10.1371/journal.pone.0271671 (PMC9307154; doi:10.1371/journal.pone.0271671)

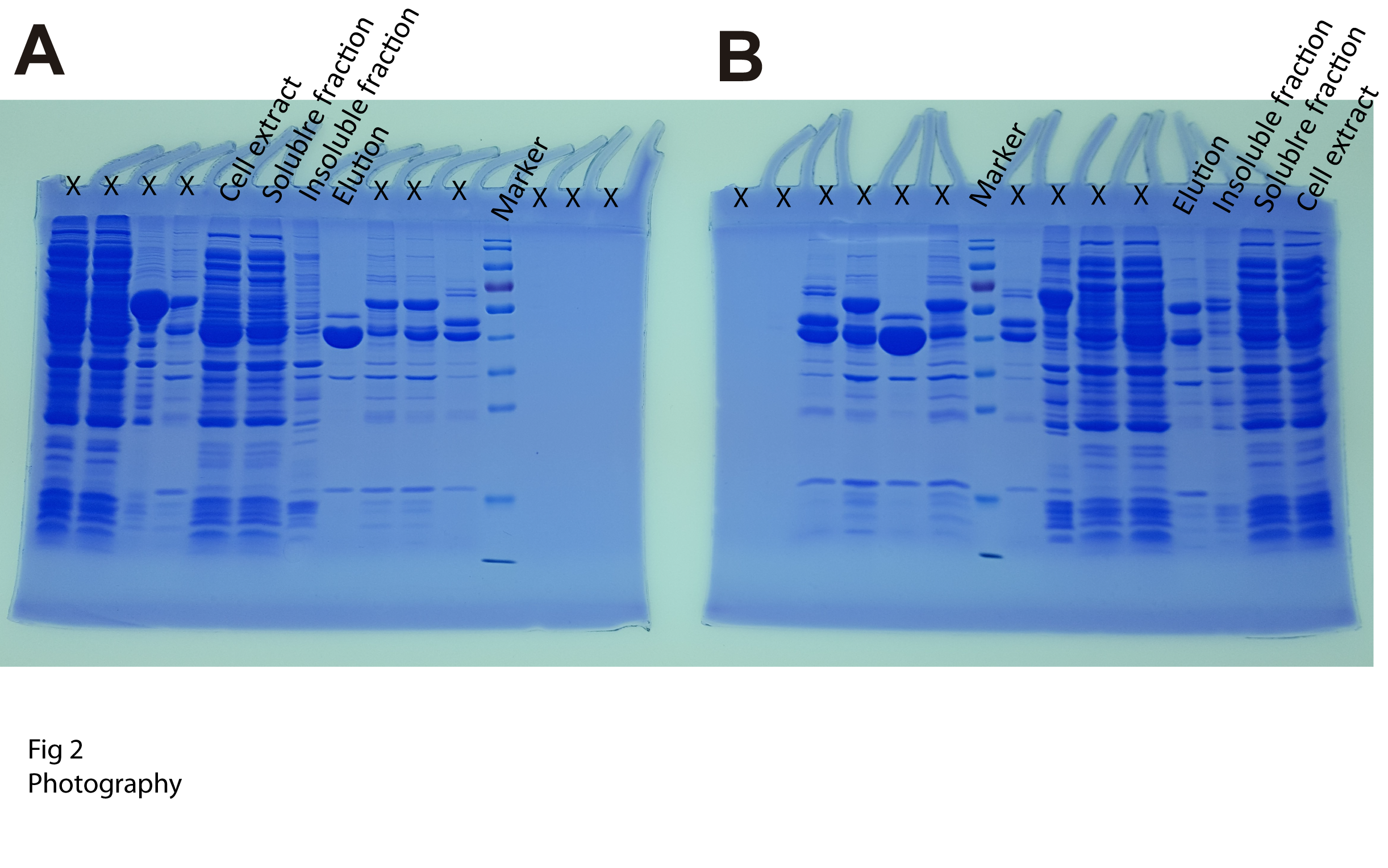

Supplement: S1 Raw image — (TIF) [file pone.0271671.s001.tif]

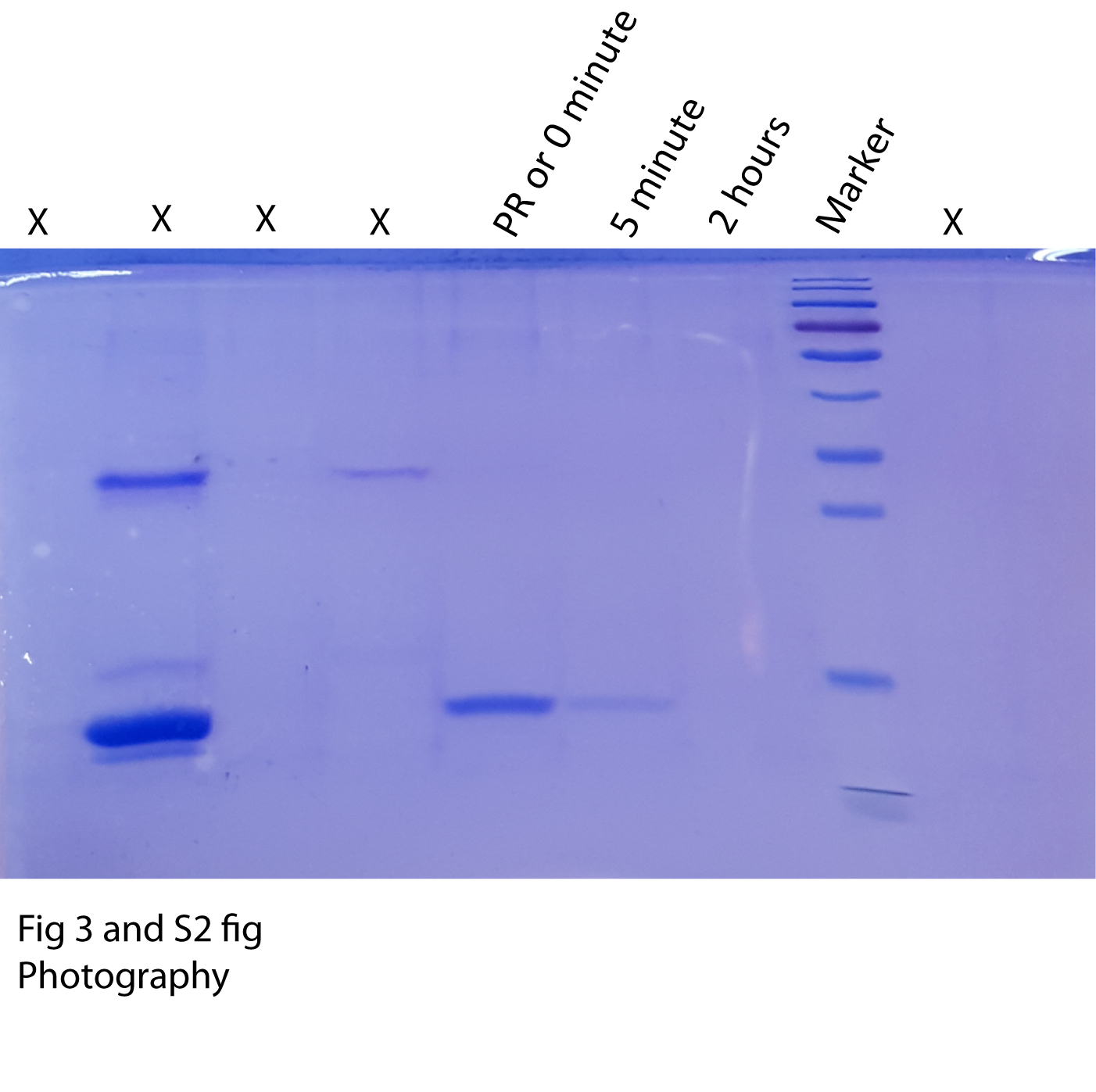

Supplement: S2 Raw image — (TIF) [file pone.0271671.s002.tif]

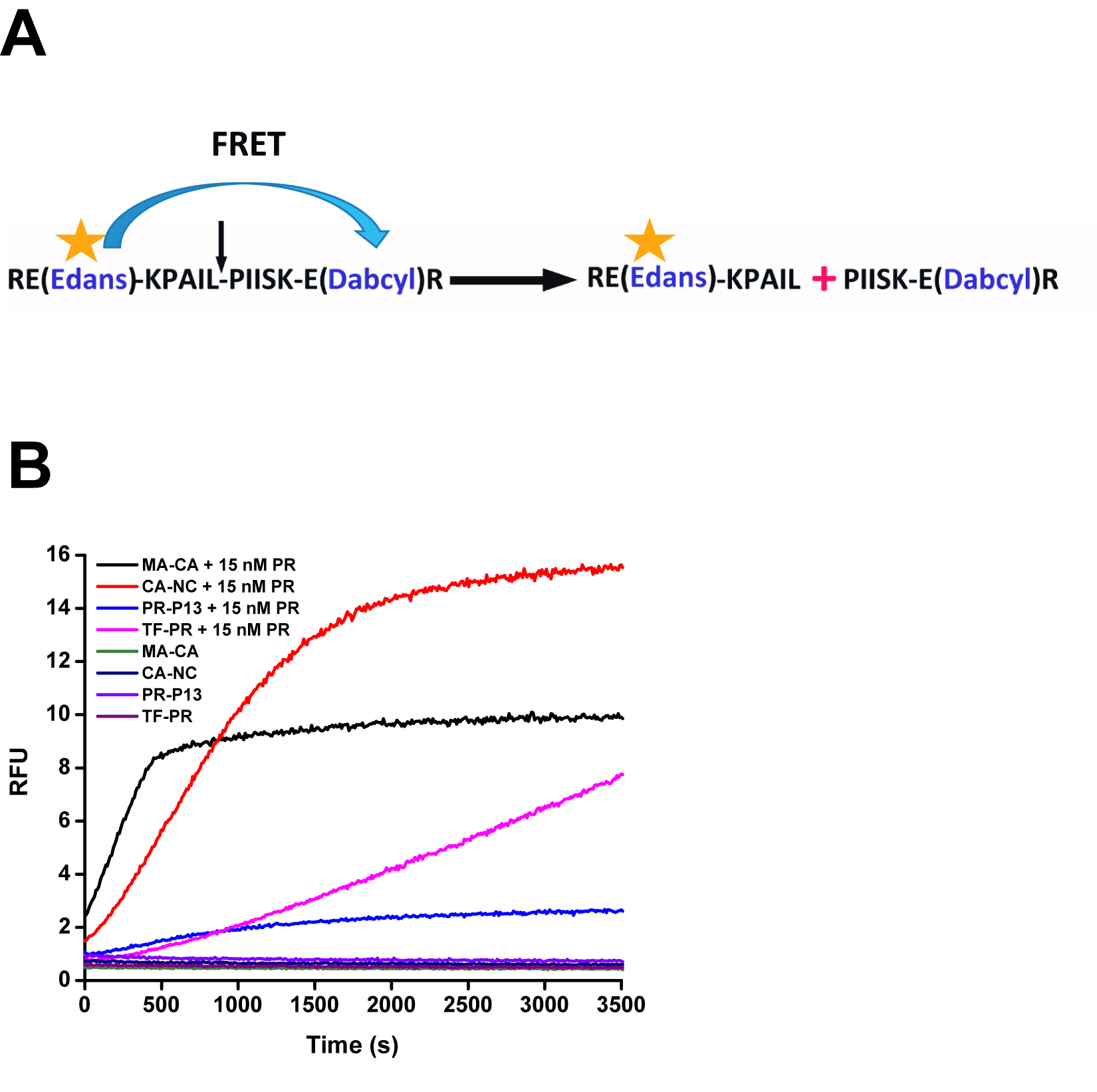

Supplement: S1 File — (ZIP) [file pone.0271671.s003.zip › Supporting Information/S1 Fig.tif]

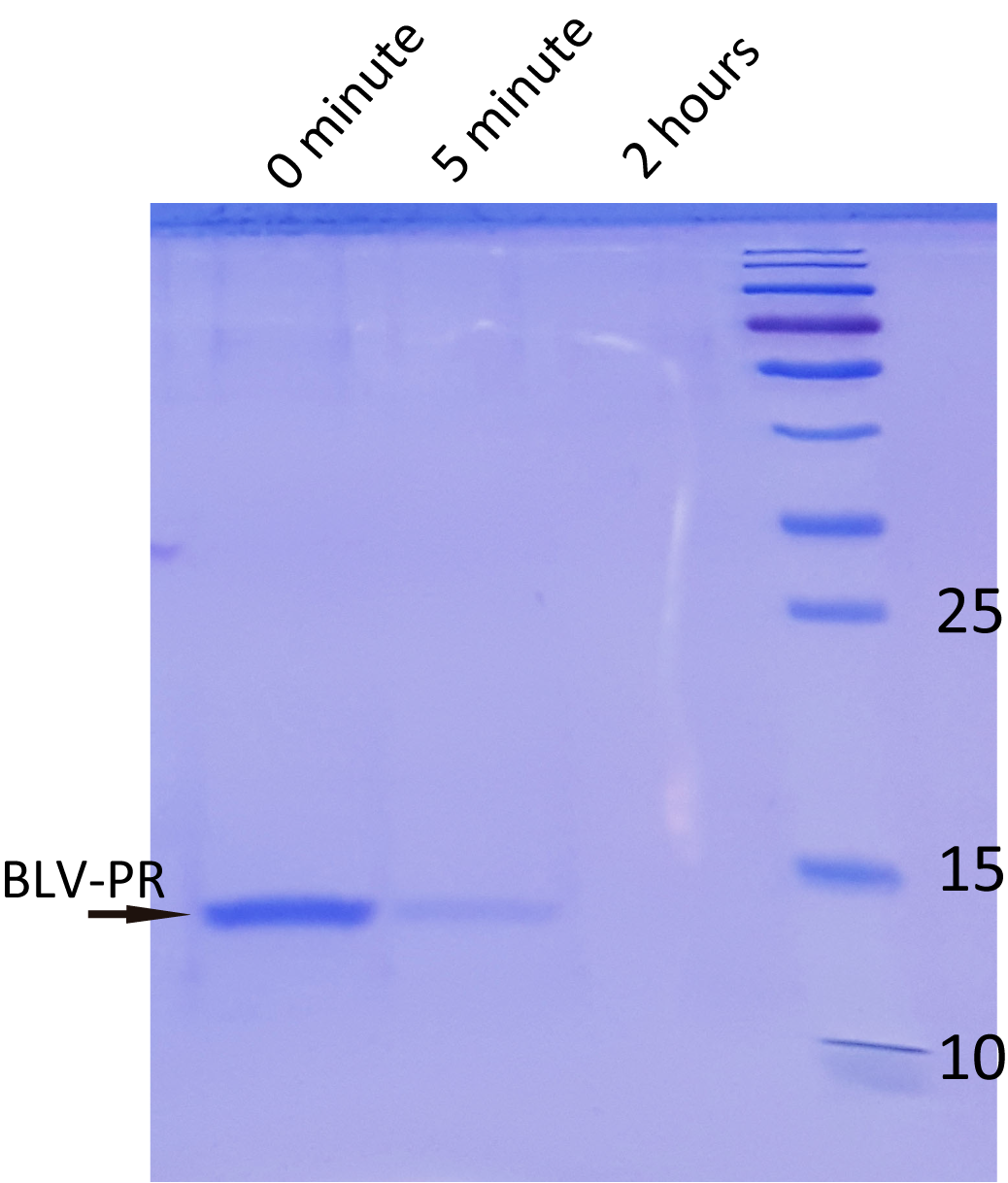

Supplement: S1 File — (ZIP) [file pone.0271671.s003.zip › Supporting Information/S2 Fig.tif]
