# Supplementary material for: Kinetics of Bovine leukemia virus aspartic protease reveals its dimerization and conformational change
Source: PLoS One. 2022 Jul 22;17(7):e0271671. doi: 10.1371/journal.pone.0271671 (PMC9307154; doi:10.1371/journal.pone.0271671)
Supplement: S1 File — (ZIP) [file pone.0271671.s003.zip › Supporting Information/Secuence MBP-pr-BLV-PR and MBP-tev-BLV-PR.docx]

Protein sequence, underlined in green and in bold the pr and tev sites are indicated, underlined in yellow immature BLV-PR and in red mature BLV-PR.

>MBP-pr-BLV-PR

MRGSHHHHHHGSSGMKTEEGKLVIWINGDKGYNGLAEVGKKFEKDTGIKVTVEHPDKLEEKFPQVAATGDGPDIIFWAHDRFGGYAQSGLLAEITPDKAFQDKLYPFTWDAVRYNGKLIAYPIAVEALSLIYNKDLLPNPPKTWEEIPALDKELKAKGKSALMFNLQEPYFTWPLIAADGGYAFKYENGKYDIKDVGVDNAGAKAGLTFLVDLIKNKHMNADTDYSIAEAAFNKGETAMTINGPWAWSNIDTSKVNYGVTVLPTFKGQPSKPFVGVLSAGINAASPNKELAKEFLENYLLTDEGLEAVNKDKPLGAVALKSYEEELAKDPRIAATMENAQKGEIMPNIPQMSAFWYAVRTAVINAASGRQTVDEALKDAQTNGSGSEN**LYFQGLIEGGLGAPQTVTPITDPLSEAELECLLSIPLARSRPSVAVYLSGPWLQPSQNQALMLVDTGAENTVLPQNWLVRDYPRIPAAVLGAGGVSRNRYNWLQGPLTLALKPEGPFITIPKILVDTFDKWQILGRDVLSRLQASISIPEEVRPPMVGVLDAPPSHIGLEHLPAPPEVPQFPLN**

>MBP-tev-BLV-PR

MRGSHHHHHHGSSGMKTEEGKLVIWINGDKGYNGLAEVGKKFEKDTGIKVTVEHPDKLEEKFPQVAATGDGPDIIFWAHDRFGGYAQSGLLAEITPDKAFQDKLYPFTWDAVRYNGKLIAYPIAVEALSLIYNKDLLPNPPKTWEEIPALDKELKAKGKSALMFNLQEPYFTWPLIAADGGYAFKYENGKYDIKDVGVDNAGAKAGLTFLVDLIKNKHMNADTDYSIAEAAFNKGETAMTINGPWAWSNIDTSKVNYGVTVLPTFKGQPSKPFVGVLSAGINAASPNKELAKEFLENYLLTDEGLEAVNKDKPLGAVALKSYEEELAKDPRIAATMENAQKGEIMPNIPQMSAFWYAVRTAVINAASGRQTVDEALKDAQTNGSGSEN**LYFQLSIPLARSRPSVAVYLSGPWLQPSQNQALMLVDTGAENTVLPQNWLVRDYPRIPAAVLGAGGVSRNRYNWLQGPLTLALKPEGPFITIPKILVDTFDKWQILGRDVLSRLQASISIPEEVRPPMVG**VLDAPPSHIGLEHLPAPPEVPQFPLN
